# Potential role of new molecular plasma signatures on cardiovascular risk stratification in asymptomatic individuals

**DOI:** 10.1038/s41598-018-23037-7

**Published:** 2018-03-19

**Authors:** Montserrat Baldan-Martin, Juan A. Lopez, Nerea Corbacho-Alonso, Paula J. Martinez, Elena Rodriguez-Sanchez, Laura Mourino-Alvarez, Tamara Sastre-Oliva, Tatiana Martin-Rojas, Raul Rincón, Eva Calvo, Jesus Vazquez, Fernando Vivanco, Luis R. Padial, Gloria Alvarez-Llamas, Gema Ruiz-Hurtado, Luis M. Ruilope, Maria G. Barderas

**Affiliations:** 1 Department of Vascular Physiopathology, Hospital Nacional de Paraplejicos (HNP), SESCAM, Toledo, Spain; 2 Cardiovascular Proteomics Laboratory and CIBER-CV, CNIC, Madrid, Spain; 3 Departament of Immunology, IIS-Fundacion Jimenez Diaz, Madrid, Spain; 4 Laboratory of Hypertension and Cardiovascular Risk, Instituto de Investigación i+12, Hospital Universitario 12 de Octubre, Madrid, Spain; 5 Ibermutuamur, Madrid, Spain; 6 Departamento de Bioquimica y Biologia Molecular I, Universidad Complutense, Madrid, Spain; 7 Departamento de Cardiologia, Complejo Hospitalario de Toledo, SESCAM, Toledo, Spain; 8 Department of Preventive Medicine and Public Health, School of Medicine, Universidad Autónoma de Madrid/IdiPAZ and CIBER in Epidemiology and Public Health (CIBERESP), Madrid, Spain; 9 School of Doctoral Studies and Research, Universidad Europea de Madrid, Madrid, Spain

## Abstract

The evaluation of cardiovascular (CV) risk is based on equations derived from epidemiological data in individuals beyond the limits of middle age such as the Framingham and SCORE risk assessments. Lifetime Risk calculator (QRisk^®^), estimates CV risk throughout a subjects’ lifetime, allowing those. A more aggressive and earlier intervention to be identified and offered protection from the consequences of CV and renal disease. The search for molecular profiles in young people that allow a correct stratification of CV risk would be of great interest to adopt preventive therapeutic measures in individuals at high CV risk. To improve the selection of subjects susceptible to intervention with aged between 30–50 years, we have employed a multiple proteomic strategy to search for new markers of early CV disease or reported CV events and to evaluate their relationship with Lifetime Risk. Blood samples from 71 patients were classified into 3 groups according to their CV risk (healthy, with CV risk factors and with a previously reported CV event subjects) and they were analyzed using a high through quantitative proteomics approach. This strategy allowed three different proteomic signatures to be defined, two of which were related to CV stratification and the third one involved markers of organ damage.

## Introduction

Cardiovascular (CV) disease represents the main cause of death worldwide^1^. CV and renal diseases develop slowly and silently, and the use of early predictors of target organ damage should be investigated and used as early as possible to avoid the progression of these diseases with significant socioeconomic benefits^2^. It is clear that the lifetime risk tool is not precise enough as one may wish to help CV risk stratification in individuals between 30–60 years^3^.

**Figure 1:**
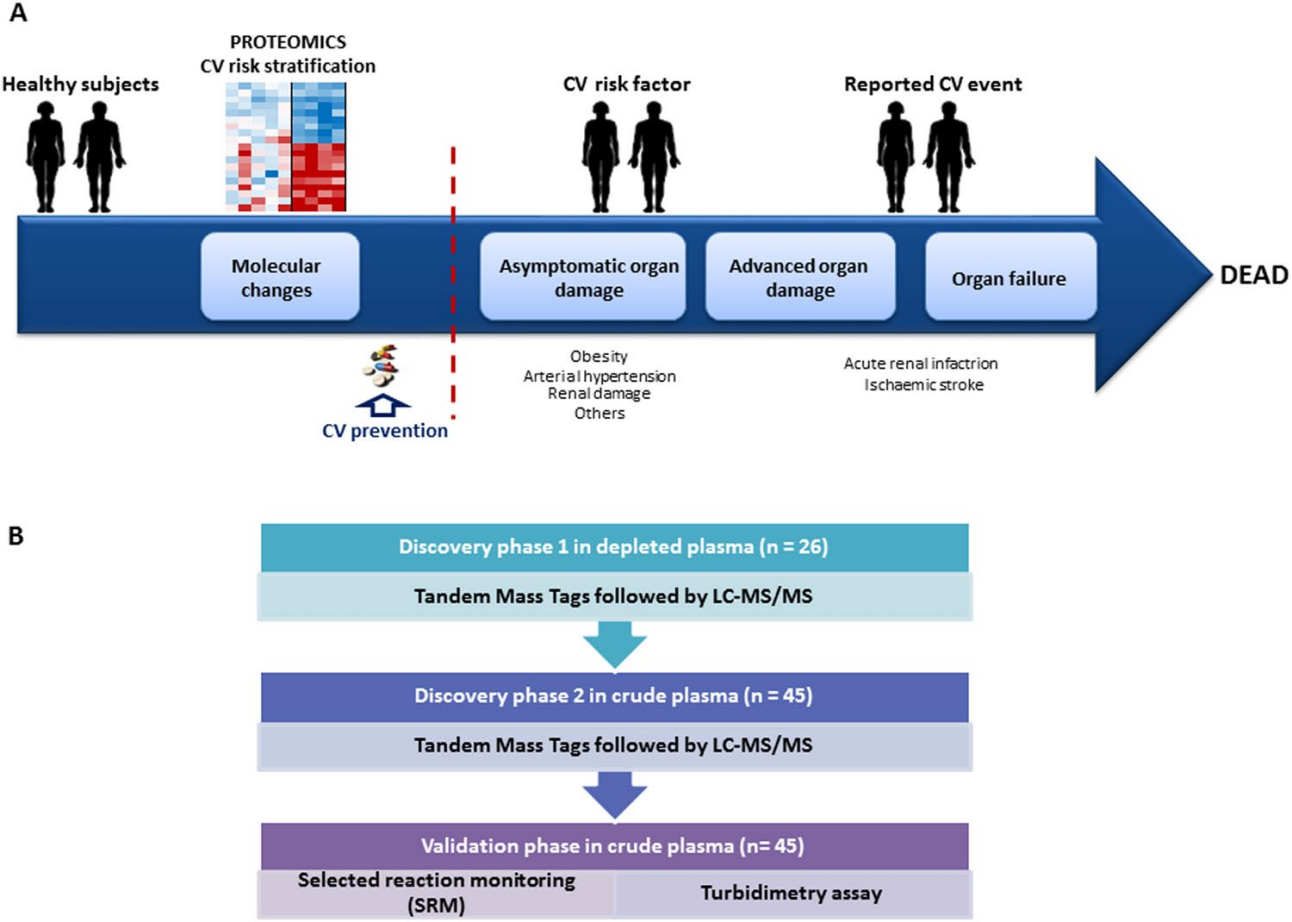
(**A**) Overview of the proteomic strategies used to stratify cardiovascular risk in individuals aged between 30–50 years old. (**B**) Experimental design consisting of two different discovery phases, one of them using depleted plasma and the other analyzing non-depleted plasma from an independent cohort of patients. Moreover, a confirmation phase was performed employing two orthogonal techniques, selected reaction monitoring (SRM) and turbidimetry.

The identification of subjects with elevated CV and renal risk not identified by Framingham nor SCORE due to their younger age would facilitate an earlier and aggressive intervention, where the concept that “the sooner the better” would complement the concept that the “lower the better” regarding the treatment and control of CV and renal risk factors. The calculator QRisk® (http://www.qrisk.org/lifetime/index.php) estimates the risk of CV disease throughout the lifetime, flagging subjects that should receive more intense interventions. However, the identification of early molecular markers could help to better identify patients at risk in a more stringent way, improving the capacity of the lifetime risk calculation. This enhancement would be useful for young patients in whom some CV risk factors are elevated (for example, arterial hypertension) (Fig. 1).

Our previous studies focused on the application of multi-omics approaches to search for molecular profiles in CV diseases, identifying protein signatures in plasma^4,5^, urine^6^ and circulating extracellular vesicles^7^ associated with cardio-renal damage. Here, we adopted a multiplexed strategy based on isobaric labeling for quantitative bottom-up proteomics to search for profiles of individuals classified according to their CV risk. This approach defined three characteristic plasma signatures that could help stratify individuals according to CV disease risk and that could identify asymptomatic young people. Our results not only provide new information about the molecular processes altered in individuals with higher CV risk but also, these proteins might help to improve CV risk estimation, thereby improving their clinical outcome.

## Results

**Figure 2:**
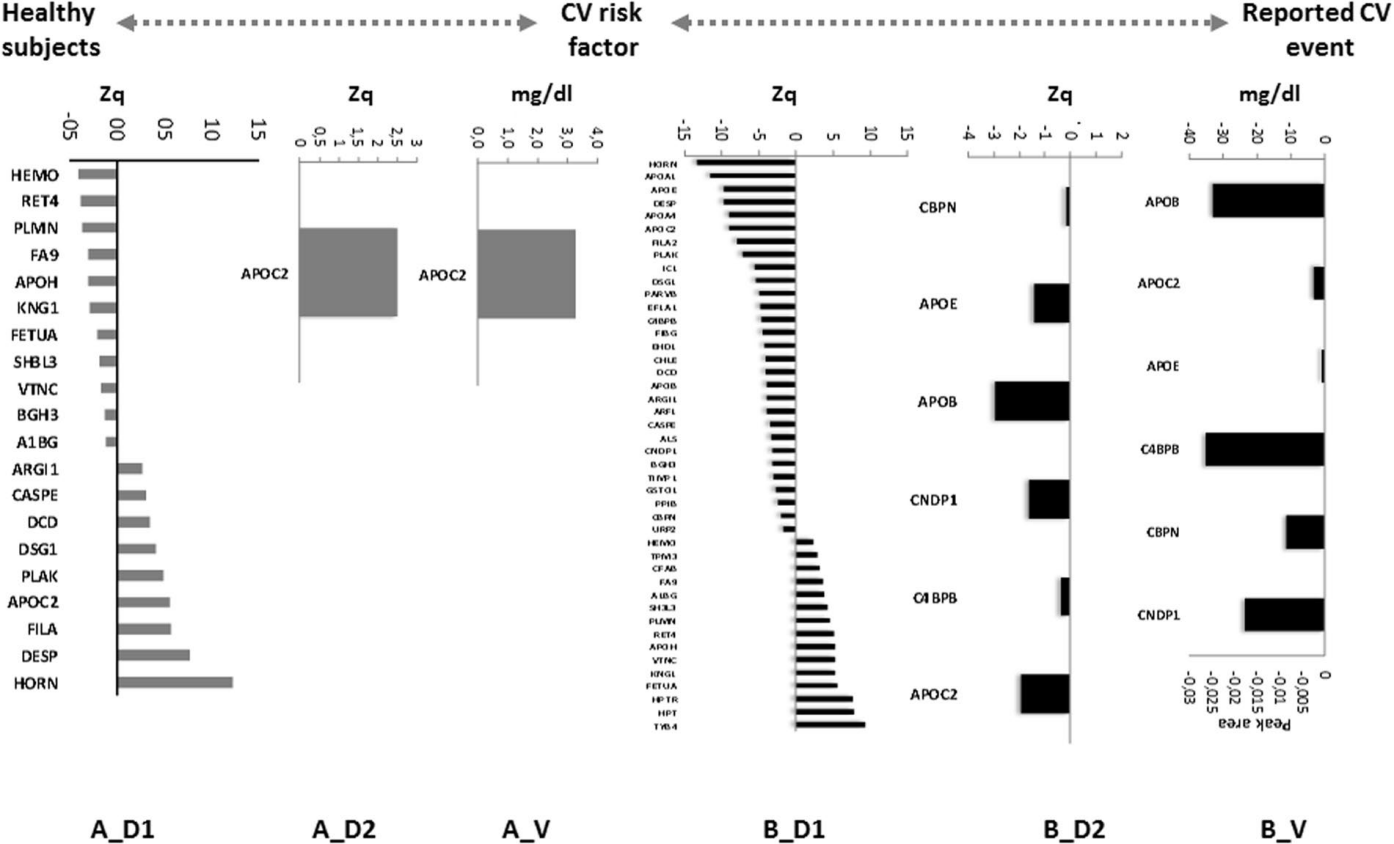
Plasma signature for CV stratification. A_D1 and B_D1) Panel of proteins in depleted plasma studied in the discovery phase 1 differentially expressed between the different groups. A_D2 and B_D2) Proteins verified in crude plasma from an independent cohort of patients in discovery phase 2. A_V and B_V) Confirmation of the proteins altered in both types of plasma when analyzed by SRM and turbidimetry. The statistical differences between the groups were calculated using a Student’s t-test.

**Figure 3:**
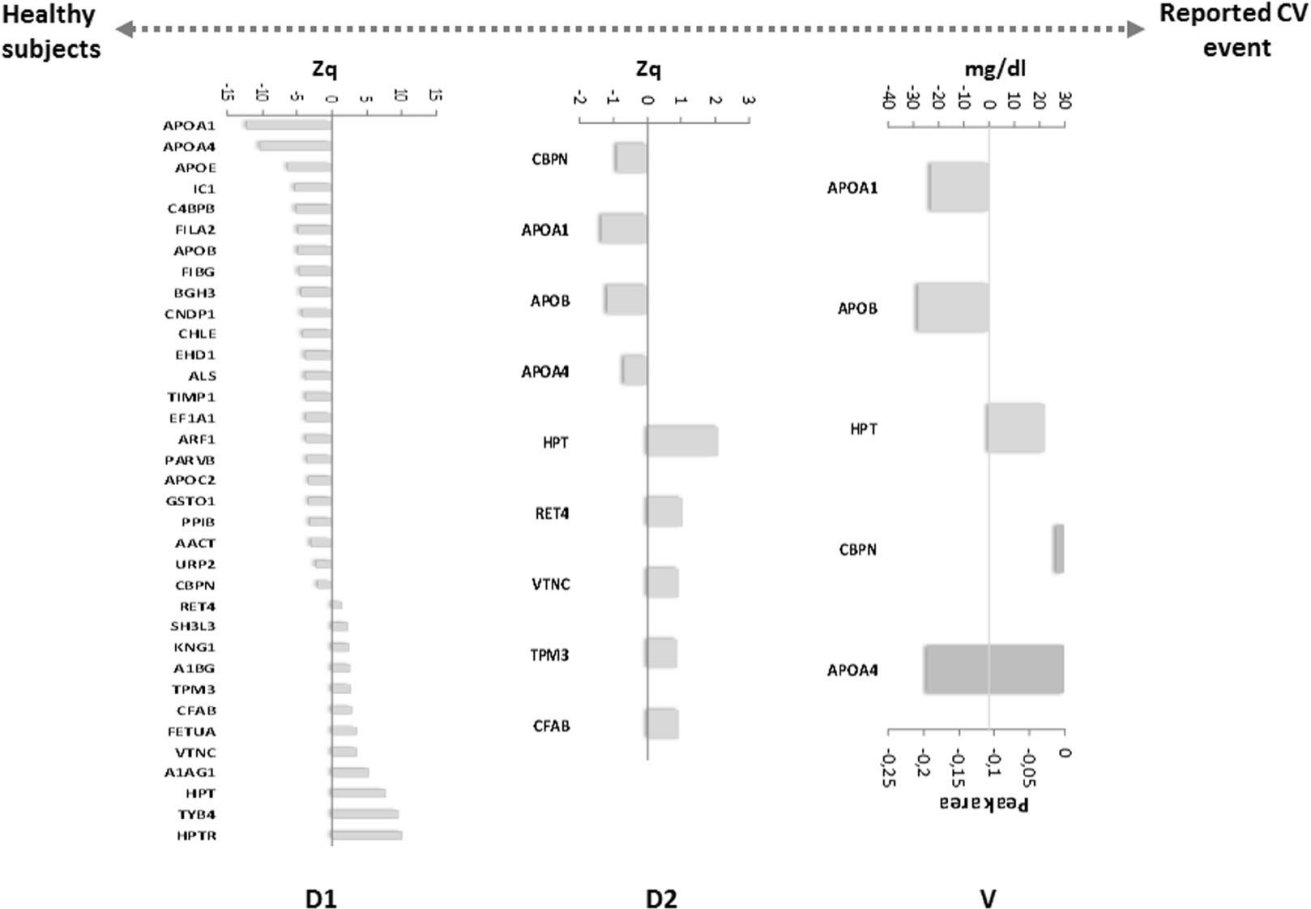
Panel of proteins that serve as markers of organ damage. (D1) Panel of 35 proteins in depleted plasma differentially expressed in patients with a reported CV event compared with healthy subjects study in discovery phase 1. (D2) 9 proteins verified in the crude plasma of an independent cohort of patients in discovery phase 2. (V) Confirmation of 5 proteins differentially expressed in both types of plasma analyzed by SRM and turbidimetry. The statistical differences between the groups were calculated using a Student’s t-test.

To identify proteomic profiles related to CV disease risk in a cohort of subjects aged 30–50 years old, we carried out a multi-proteomics study. These analyses allowed us to carry out a deep proteomics analysis, where2067 proteins were quantified, of them 47 were differentially expressed (Table S1). A panel of 20 proteins was altered in patients with CV risk factors compared with healthy controls (Fig. 2A_D1). In addition, a set of 44 proteins having different expression was found in patients with CV event in comparison with CV risk factors(Fig. 2B_D1) and finally, another panel of 35 proteins was differentially expressed in individuals with a CV event compared to healthy controls (Fig. 3-D1). To verify these alterations in an independent cohort of subjects, a second TMT experiment was performed using non-depleted plasma, a sample most used in a clinical setting. In the comparison of the healthy subjects with the CV risk factor group, we found the same trend for the APOC2protein (Fig. 2A_D2), and in patients with a CV event, we observed the same expression of 6 proteins compared with the CV risk factor group: CPBN, APOE, APOB, CNDP1, C4BPB, and APOC2 (Fig. 2B_D2). In addition, 9 proteins were altered in patients with a CV event compared with healthy subjects: CBPN, APOA1, APOB, APOA4, HPT, RET4, VTNC, TPM3, and CFAB (Fig. 3-D2).

Of the differentially expressed proteins found in both types of plasma, 5 proteins were measured by turbidimetry, an assay commonly employed in clinical practice, and 8 proteins were analyzed by SRM, a clinically relevant method for quantitative analysis.

### CV disease stratification

**Figure 4:**
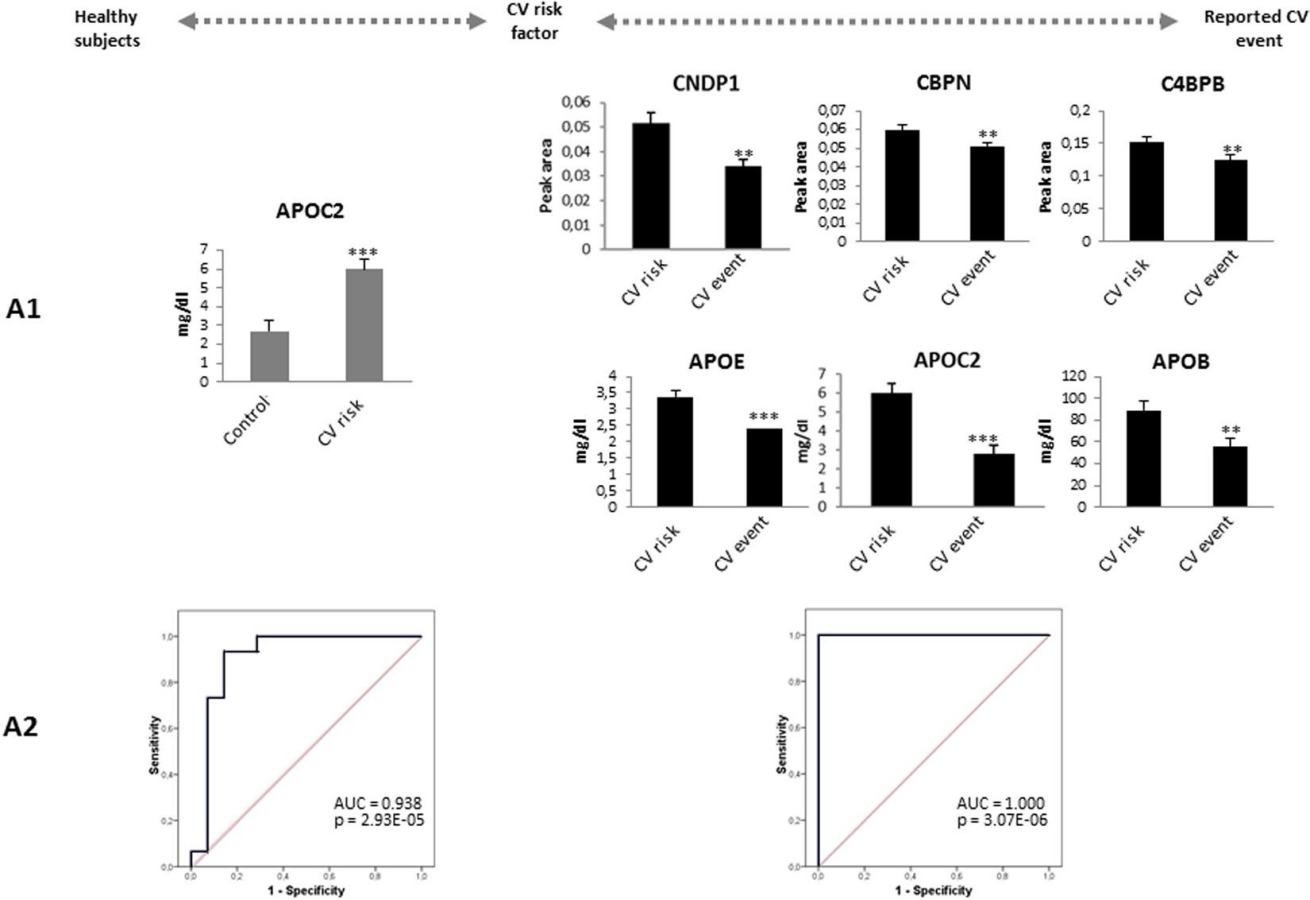
(A1) Confirmation of proteins related to CV stratification by SRM and turbidimetry. (A2) ROC curves for the classification of patients with a CV risk factor compared to healthy subjects, and patients with a reported CV event with respect to individuals with CV risk factors. Statistical significance was accepted at: *p < 0.05, **p < 0.01, ***p < 0.001.

A comparative analysis was carried out between the groups stratified according to CV disease risk. We first confirmed the increased APOC2 in individuals with CV risk factor relative to the healthy controls (Fig. 2A_V and Fig. 4-A1). These results were reinforced by the ROC analysis, showing that the area under the curve (AUC) can discriminate these two groups (AUC = 0.938, p-value = 2.93E-0.5: Fig. 4-A2). On the other hand, we found a panel of 6 proteins whose levels were lower in individuals with a reported CV event: CNDP1, CBPN, C4BPB, APOE, APOC2, and APOB (Fig. 2B_V and Fig. 4-A1). In addition, the ROC analysis obtained with this protein panel classified these patients perfectly through their protein levels (Fig. 4-A2).

### Panel of protein markers of organ damage

**Figure 5:**
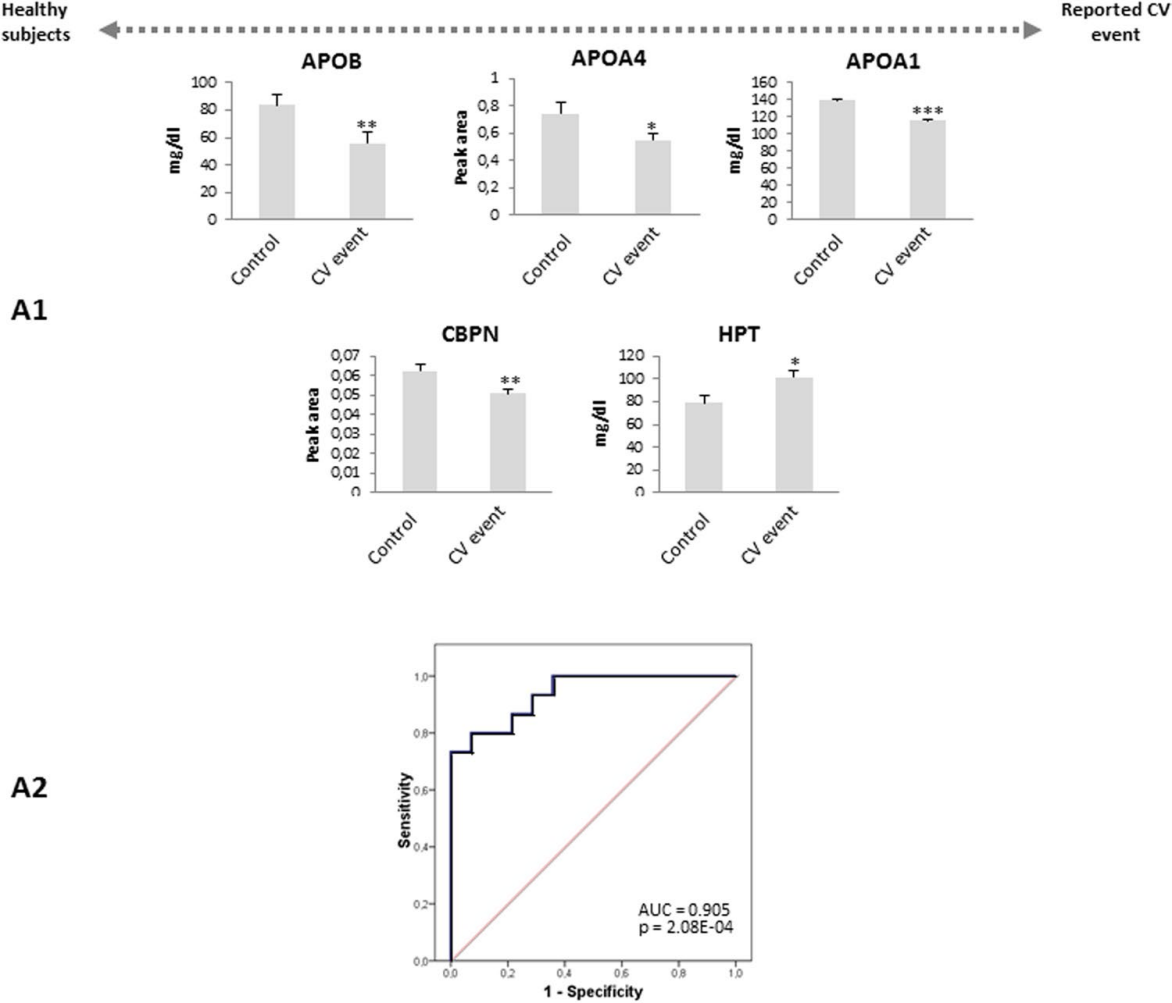
(A1) Confirmation of 5 proteins related to organ damage confirmed by SRM and turbidimetry. (A2) The ROC curves to distinguish the patients with a reported CV event from the healthy subjects. Significant changes are indicated as: *p < 0.05, **p < 0.01, ***p < 0.001.

**Figure 6:**
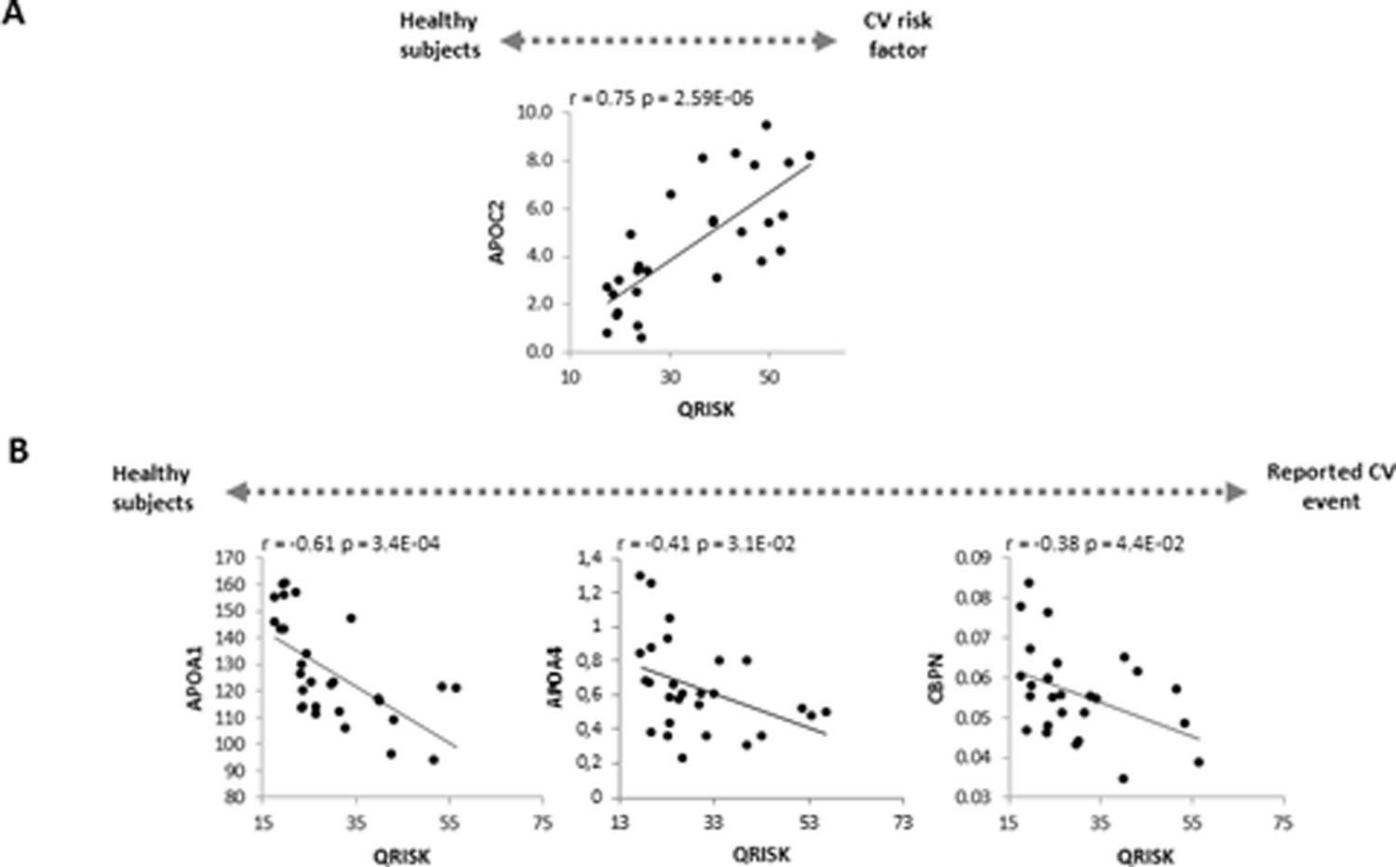
Correlation analysis of the confirmed proteins with QRISK. (**A**) Correlation between APOC2 with QRISK in healthy subjects and individuals with a CV risk factor. (**B**) Correlations between APOA1, APOA4 and CBPN in healthy subjects and patients with a reported CV event.

A group of 5 proteins were confirmed in the comparison between subjects with a CV event and healthy controls, suggesting the potential value of this panel as markers of organ damage: APOB, APOA4, APOA1, CBPN, and HPT (Fig. 3-V and Fig. 5-A1). The ROC analysis for this panel showed good sensitivity and specificity (AUC = 0.905, p-value = 2.08E-04: Fig. 5-A2). To assess if there were relationships between these proteins with the QRISK score, we performed a correlation analysis. In terms of the evolution of healthy controls towards a CV disease risk factors, we found a positive correlation of APOC2 and QRISK between the two groups (r = 0.75, p-value = 2.59E-06), highlighting the value of this protein in assessing the evolution of CV disease (Fig. 6A). Conversely, negative correlations existed between APOA1(r = −0.61, p-value = 3.4E-04), APOA4 (r = −0.41, p-value = 3.1E-02) or CBPN (r = −0.38, p-value = 4.4E-02) and QRISK, both in healthy as well as in individuals with CV event, these proteins representing potential markers to evaluate organ damage (Fig. 6B).

**Figure 7:**
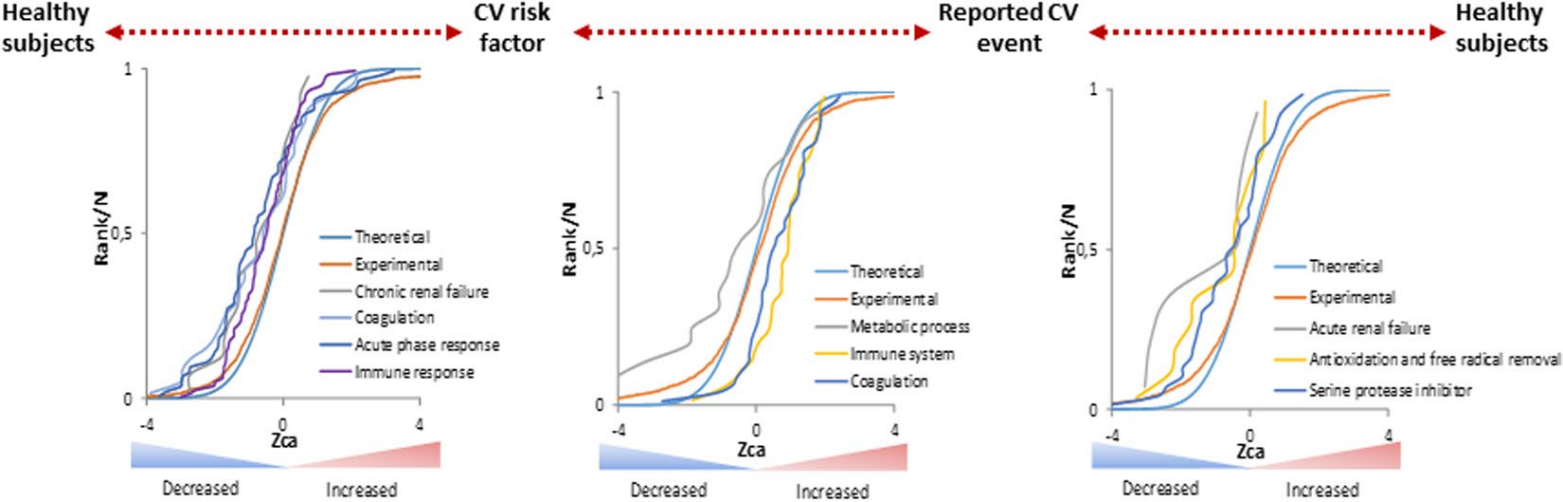
Altered functional categories in patients with CV risk factors and a reported CV event evident using the System Biology Triangle (SBT) model. Distribution of the standardized variable of coordinated proteins (Zca) identifies coordinated protein changes. Categories related to chronic renal failure, coagulation, acute phase response and the immune system in patients with CV risk factors *versus* healthy subjects, and the cluster of differentially altered proteins related to metabolic process, complement-mediated immunity and coagulation in patients with a reported CV event *versus* patients with a CV risk factors. Altered categories related to acute renal failure, antioxidation and free radical removal and serine protease inhibitor in patients with a reported CV event *versus* healthy subjects.

The SBT algorithm allowed to identify 4 down-regulated clusters related to renal failure, coagulation, acute phase and immune response in the group of CV risk factor vs healthy (Fig. 7A and Table 1); and 3 clusters in individuals with a CV event *versu*s CV risk factors, whereby proteins associated with the coagulation and immune response are enhanced, while those in the metabolic process are dampened (Fig. 7B and Table 1). In addition, there is a focus on organ damage when we compared patients with a CV event with healthy subjects, involving a decrease in the categories related to renal failure, free radical removal, and serine protease inhibition (Fig. 7C and Table 1).

**Table 1:** Categories altered between different groups of study. Data are expressed in the form of standardized variable of coordinated proteins (Zca) and False Discovery Rate of category (FDRca).

Functional Categories	*Z*ca	*FDR*ca
**CV risk factor** ***versus*** **Healthy controls**		
Acute Phase Response Signaling	−5.392	8.00E-06
Chronic renal failure	−3.053	3.80E-02
Coagulation	−4.785	1.43E-04
Signaling in Immune system	−4.205	1.34E-03
**Reported CV event** ***versus*** **CV risk factor**		
Complement-mediated immunity	3.890	5.30E-03
Metabolic process	−3.737	8.40E-03
Coagulation	3.271	3.70E-02
**Reported CV event** ***versus*** **Healthy controls**		
Acute renal failure	−3.332	3.40E-02
Antioxidation and free radical removal	−4.647	3.30E-04
Serine protease inhibitor	−3.057	7.20E-02

## Discussion

It has been hypothesized that an adequate treatment could translate simultaneously in CV and renal protection.The discovery of new early predictors of cardio-renal disease detected before the appearance of target organ damage is required for the purpose of an early prevention^8^. Importantly, only 5–8% of adult’s older than 50 years of age have been shown to present an optimal CV risk profile associated with a low risk of developing CV disease with advancing age^9^. Hence, an appropriate prevention strategy that focuses on the minimization of the impact of CV and renal disease must start at earlier ages. The identification of signatures with potential value in the stratification of CV risk in asymptomatic individuals would allow the adoption of better prevention strategies for CV protection. Here, we present the first proteomic study that set out to identify markers that potentially serve to classify middle aged individuals into different groups according to their CV disease risk (healthy subjects, subjects with clustering of CV risk factors and subjects with establish CV disease). Such markers should help to adopt better prevention strategies for CV disease protection in asymptomatic individuals, a particularly important clinical issue. Some of the proteins identified in this work (haptoglobin, fibrinogen, alpha 1-acid glycoprotein, apoliprotein AI should have been removed by MARS 14, but it is important to note that not all of the targeted proteins were captured equally by the MARS-14 column (whereas haptoglobin 99%, fibrinogen 76% and apolipoprotein AI only up to 47% efficiency by the MARS-14 column) even so, our methodology is totally correct for the purpose of our investigation^10^.

Regarding the signature of CV risk stratification, the turbidimetry assay confirmed the increase of APOC2 in patients with CV disease risk factors relative to healthy subjects. APOC2 is a component of chylomicrons, VLDL and HDL, and the relationship between cholesterol and atherosclerotic CV disease is well established^11^. High levels of APOC2 alter HDL distribution, suggesting a role for these proteins in risk of CV disease^12^. Moreover, ROC curve reflects the potential value of APOC2 to identify individuals that are at high risk of developing CV disease in a short period of time.

A panel of 6 down-regulated proteins was confirmed in patients with CV event relative to those with CV risk factors. Regarding to apolipoproteins, APOE and APOC2 are involved in processes related to lipid metabolism, blood coagulation and inflammation. APOB can serve as a direct measure of the atherogenic lipoprotein particles in circulation^13^. An association of APOE, APOC2 and APOB with CV disease has been reported previously^14^. Thus, we think that measuring this panel in clinical practice to monitor patients with a high risk of CV disease could improve the prediction of this disease. This predictive signature also includes two proteins characterized by their carboxypeptidase activity. CNDP1 is a metallopeptidase that hydrolyze carnosine, a radical oxygen species scavenger and natural inhibitor of the angiotensin converting enzyme that diminishes blood pressure^15^. Hence, reduced CNDP1 might prevent the formation of the active form of carnosine, resulting in enhanced oxidative stress and possibly, affecting blood pressure control. On the other hand, CBPN possesses anti-inflammatory activity and it plays a pro-coagulant role during fibrin clot formation^16^.

C4BPB is a protein involved in the regulation of the complement^17^ and it influences coagulation^18^. Decreased levels of C4BPB may be associated with the enhanced pro-coagulant activity in high CV risk patients.

We showed a third panel of 5 proteins that serve as indicators of end-organ damage. In addition to a decrease in APOB, patients with a reported CV event displayed two down-regulated apolipoproteins. APOA4 circulates freely or in association with chylomicrons and HDL, and a decrease in plasma has been correlated with coronary heart disease^19^. Additionally, it inhibits lipid peroxidation^20^ and thus, decreased levels may reflect an increase in oxidative stress in these individuals. Alternatively, APOA1 has an important athero-protective function thanks to its anti-inflammatory role and its influence on the recruitment of monocytes/macrophages to local sites of inflammation^21^. It has also been proposed to have anti-oxidant activity^22^. APOA1 has been proposed as a potentially promising therapeutic targetto treat CV disease by augmenting HDL cholesterol efflux and hence, reducing the risk of CV disease^23^. Conversely, an increase in HPT was found, an acute-phase protein that fulfils a role in neutralizing oxidative damage. Moreover, the systems biology analysis provides additional information about the biological processes altered as CV disease progresses.

Our data have the limitation of the small number of subjects. Further studies are needed which could help us to demonstrate the application of our promising results as preventive strategy which effectively protects subjects with a particular protein profile. These studies are needed to secure the great potential of this subset of proteins in early detection of asymptomatic individuals with a higher risk of developing CV diseases.

In conclusion, the relevance of our findings consists on the description of initial new markers for early detection of asymptomatic individuals at a higher risk of developing the clinical consequences of CV diseases. This may facilitate an adequate stratification of early CV risk in those patients with clustering of CV risk factors. In this sense longitudinal population based studies are required. Simultaneously, these markers allow the identification of patients with a significantly increase lifetime risks representing a much more accurate form of identifying middle aged subjects at risk of developing CV disease and allowing a prompt and adequate intervention. Accordingly, future preventive strategies should be based not only on the established algorithms and traditional markers but also, on the use of these proteomic profiles that represent a new approach to predict the evolution of these individuals.

## Methods

### Patients selection and sample preparation

Plasma samples were obtained from individuals aged 30–50 years old, stratified in accordance to their CV risk. A total of 71 subjects were classified into the following subgroups: (a) healthy controls (n = 24); (b) patients with CV disease risk factors (arterial hypertension or patients treated with anti-hypertensive medications, glycaemia (glucose in blood >100 mg/dl) and/or metabolic syndrome: n = 23); and (c) patients with a previously reported CV event (acute myocardial infarction, acute coronary syndrome or angina pectoris) (in the past 4 years, n = 24). Baseline demographic data were recorded at the time of recruitment. All subjects included in the study were screened with detailed medical history, physical examination and biochemical profile. The subjects were asked to participate in the study. In case of acceptance, they signed the informed consent and 7 mL of blood were withdrawn for analysis and introduced in EDTA-prepared collection tubes (Venoject, Terumo Europe). Samples were immediately taken to our laboratory to prevent sample degradation (less than 3 hours). Finally, samples were centrifuged at 3500 *g* (5810 R, Eppendorf) for 10 min at 4 °C and the resulting plasma was aliquoted in batches of 500 μl and stored at −80 °C until proteomic analysis. The baseline characteristics of the patients are shown in Table 2. The study was carried out according to the recommendations of the Declaration of Helsinki and it was approved by the local ethics committee (Complejo Hospitalario de Toledo, Hospital Virgen de la Salud).

**Table 2:** Baseline characteristics of the patients recruited for the study. Data are expressed as mean ± standard deviation (SD) or percentages (%). Statistical differences between groups of patients were calculated by one-way ANOVA (p < 0.05 was considered significantly).

Discovery phase	Control (n = 10)	CV risk factor (n = 8)	CV event (n = 8)	P-value
Age (years)	44 ± 5	44 ± 6	46 ± 4	0.700
Sex (male), %	60	50	50	0.884
Current smoking, %	0	25	50	0.043
Total cholesterol (mg/dl)	187 ± 25	206 ± 39	145 ± 40	0.008
HDL cholesterol (mg/dl)	72 ± 18	53 ± 13	44 ± 10	0.001
LDL cholesterol (mg/dl)	101 ± 24	131 ± 36	77 ± 32	0.010
Triglycerides (mg/dl)	73 ± 26	139 ± 130	124 ± 67	0.222
Glycaemia (mg/dl)	76 ± 7	104 ± 48	94 ± 30	0.280
Uric acid (mg/dl)	4.8 ± 1.6	5 ± 1	5.5 ± 1.6	0.620
Metabolic syndrome, %	0	25	38	0.171
eGFR (ml/min/1.73 m^2^)	91 ± 9	97 ± 22	103 ± 34	0.585
Systolic blood pressure (mmHg)	113 ± 10	131 ± 5	125 ± 19	0.017
Diastolic blood pressure (mmHg)	72 ± 8	84 ± 10	78 ± 9	0.034
Antihypertensives, %	0	25	13	0.354
Lipid-lowering agents, %	0	13	13	0.622
QRISK	22 ± 5	33 ± 9	27 ± 6	0.004
**Confirmation phase**	**Control (n** **=** **14)**	**CV risk factor (n** **=** **15)**	**CV event (n** **=** **16)**	**P-value**
Age (years)	42 ± 5	45 ± 5	45 ± 5	0.131
Sex (male), %	21	80	94	6.20E-05
Current smoking, %	21	27	56	0.094
Total cholesterol (mg/dl)	198 ± 47	215 ± 39	145 ± 41	0.0001
HDL cholesterol (mg/dl)	69 ± 18	41 ± 16	39 ± 9	1.18E-06
LDL cholesterol (mg/dl)	109 ± 40	139 ± 34	82 ± 40	6.90E-04
Triglycerides (mg/dl)	92 ± 46	221 ± 80	113 ± 67	5.18E-06
Glycaemia (mg/dl)	80 ± 9	95 ± 22	100 ± 15	3.90E-03
Uric acid (mg/dl)	4.5 ± 1.1	6.7 ± 1.8	5.8 ± 1.7	1.99E-03
Metabolic syndrome, %	0	73	6	1.77E-13
eGFR (ml/min/1.73 m^2^)	95 ± 12	83 ± 9	94 ± 17	0.032
Systolic blood pressure (mmHg)	112 ± 9	138 ± 14	121 ± 20	1.30E-04
Diastolic blood pressure (mmHg)	70 ± 9	90 ± 9	76 ± 12	1.76E-06
Antihypertensives, %	0	40	44	0.016
Lipid-lowering agents, %	0	27	38	0.042
QRISK	23 ± 9	44 ± 9	36 ± 10	1.38E-06

### Quantitative proteomic analysis

#### Sample preparation

For the proteomics analysis carried out in the discovery phase, plasma samples were depleted of the 14 most abundant proteins by HPLC on MARS-14 columns (Agilent). The protein concentration in the non-retained fractions was determined by using a Direct Detect IR spectrometer (Millipore).

#### Protein digestion and isobaric labelling

For the quantitative differential LC-MS/MS analysis using isobaric tags (TMT 10-plex), about 100 µg of total protein was digested by the FASP protocol described previously^24^, with minor modifications. Samples were denatured by boiling for 5 min in the presence of 0.2% SDS and 50 mM iodoacetamide (IAA) and after incubating in the dark for 30 min at room temperature, the samples were diluted in 7 M urea in 0.1 M Tris-HCl (pH 8.5: UA buffer) and loaded onto 10 kDa centrifugal filter devices (NanoSep 10 k Omega, Pall Life Sciences). The buffer was replaced by washing the filters with UA buffer and the proteins were then reduced for 30 min with 10 mM TCEP (Tris(2-carboxyethyl) phosphine hydrochloride, Pierce), washed with 50 mM Hepes buffer, and alkylated for 20 min in the darkin 50 mM MMTS (methyl methanethiosulfonate: Pierce) in UA. The excess alkylating reagent was eliminated by washing three times with UA and three further times with 50 mM ammonium bicarbonate. The proteins were digested overnight at 37 °C with modified trypsin (30:1 protein:trypsin (w/w) in 50 mM ammonium bicarbonate: Promega). The resulting peptides were twice eluted by centrifugation with 50 mM ammonium bicarbonate and 0.5 M sodium chloride. Trifluoroacetic acid (TFA) was added to a final concentration of 1% and the peptides were desalted onto C18 Oasis-HLB cartridges and dried for further analysis.

For stable isobaric labelling, the resulting tryptic peptides were dissolved in 100 mM Triethylammonium bicarbonate (TEAB) buffer, and the peptide concentration was determined by measuring the amide bonds with the Direct Detect system (Millipore). Equal amounts of each peptide sample were labelled using the 10-plex TMT Reagents (Thermo Fisher) according to the manufacturer’s protocol. The peptides were labelled with the TMT reagents previously reconstituted with 70 μl of acetonitrile (ACN) and after incubation at room temperature (RT) for 2 h, the reaction was stopped by adding 0.5% TFA for 30 min. The samples were concentrated in a Speed Vac, desalted onto C18 Oasis-HLB cartridges and dried for further analysis. To increase proteome coverage, TMT-labeled samples were fractionated by high-pH reverse phase chromatography (High pH Reversed-Phase Peptide Fractionation Kit: Pierce) and concentrated as before.

To analyze the non-depleted plasma samples in the confirmation phase, samples were denatured by boiling for 5 min in 0.2% SDS and 10 mM TCEP, diluted in UA buffer and loaded onto 10 kDa centrifugal filter devices (NanoSep 10k Omega: Pall Life Sciences). The buffer was replaced by washing filters with UA, and the proteins were then alkylated with 50 mM IAA for 30 min in the dark, before they were digestion with trypsin and processed as indicated above.

#### Protein identification and quantitation

Labelled peptides were analyzed by LC-MS/MS using a C-18 reversed phase nano-column (75 µm I.D. × 50 cm, 2 µm particle size, Acclaim PepMap RSLC, 100 C18: Thermo Fisher Scientific) and a continuous acetonitrile gradient consisting of: 0–30% B for 360 min, 50–90% B in 3 min (A = 0.1% formic acid; B = 90% ACN, 0.1% formic acid -FA). A flow rate of 200 nL/min was used to elute peptides from the nano-column to an emitter nanospray needle for real time ionization and peptide fragmentation on an Orbitrap Fusion mass spectrometer (Thermo Fisher). An enhanced FT-resolution spectrum (resolution = 70,000) followed by the MS/MS spectra from the most intense parent ions were analyzed in the chromatography run. Dynamic exclusion was set at 40 s. For peptide identification, all spectra were analyzed with Proteome Discoverer (version 2.1.0.81, Thermo Fisher Scientific) using SEQUEST-HT (Thermo Fisher Scientific).

To search the Uniprot database containing all sequences from human and contaminants (May 14^th^, 2016; 70611 entries), the parameters selected were: trypsin digestion with 2 maximum missed cleavage sites; precursor and fragment mass tolerances of 2 Da and 0.02 Da, respectively; TMT modifications at N-terminal and Lys residues as fixed modifications, and methionine oxidations as dynamic modification. Related to cysteine residues, carbamidomethyl cysteine and MMTS modified-cysteines were selected as dynamic modifications for the discovery phase, while carbamidomethyl cysteine was selected as fixed modification in the confirmatory phase. Peptide identification was performed using the probability ratio method^25^ and the false discovery rate (FDR) was calculated using inverted databases and the refined method^26^, with an additional filter for precursor mass tolerance of 15 ppm^27^. The peptides identified had a FDR ≤1% and only these peptides were used to quantify the relative abundance of each protein from reporter ion intensities and for statistical analysis of the quantitative data, the WSPP statistical model described previously was used^28^. In this model the protein log2-ratios are expressed as standardized variables, i.e.: in units of standard deviation according to their estimated variances (Zq values).

#### Functional protein analysis

Functional analysis of the whole set of quantified proteins was performed analyzing coordinated protein responses in quantitative proteomics experiments, the systems biology triangle (SBT)^29^. This algorithm correlates the performance of protein groups within a biological process with their quantitative behavior. Variations in the abundance of functional categories were visualized by comparing the cumulative frequency plots of the standardized variable with that of the normal distribution, as performed previously^30^.

### Turbidimetry

This analytical technique is used to measured scattered light and the method is based on mixing anti-human antibodies with the samples assayed to form insoluble complexes. These complexes cause a change in absorbance (dispersion) that it is proportional to the protein concentration and that can be quantified by comparison with a calibrator of known protein concentration. The reagents used included a diluent buffer (Tris 20 mmol/L, PEG 8000, pH 8.3, Sodium azide 0.95 g/L) and an antiserum (goat serum reactive to the specific protein analyzed). It is recommended that a calibrator and a quality control are used. The samples were analyzed in a chemical analyzer Mindray BS200E (Bio-Medical Electronics Co., Ltd) using a wavelength of 340 nm. Plasma samples were mixed with the diluent and the basal absorbance was measured before the samples were incubated for 2 minutes with the corresponding antibody and the protein was quantified.

### SRM (Selected Reaction Monitoring)

#### SRM design

The following proteins were analyzed by SRM: C4BPB (P20851), CFAB (P00751), RET4 (P02753), VTNC (P04004), CNDP1 (Q96KN2), CBPN (P15169), TPM3 (P06753) and ApoA-IV (P06727).Proteotypic peptides were selected by *in silico* digestion using MRM Pilot software (ABsciex), excluding peptides containing methionine residues. SRM-MS quantification was based on the best peptide among the peptide candidates detected for each protein. The selected peptides were then prepared as SpikeTides™_L for relative quantification: heavy-isotope labeled Arg (13C6, 15N4) and Lys (13C6, 15N2: JPT Peptide Technologies, Berlin, Germany). Three to four transitions (Q1 m/z to Q3 m/z) were programmed for each peptide according to Table S2.

#### SRM Sample preparation

The plasma (1 µL) was diluted in 20 µL of a 1:1 100 mM ammonium bicarbonate/trifluoroethanol solution. Cysteine residues were then reduced for 30 min at 56 °C by adding 10 mM D-L-dithiothreitol (DTT). Sulfhydryl groups were alkylated in the dark at RT for 30 min with 14 mM IAA and the excess IAA was neutralized for 30 min at RT with 10 mM DTT with 50 mMammonium bicarbonate in a final volume of 100 µL. The sample was digested overnight at 37 °C with 1 μg of sequencing grade-modified trypsin (TCPK Trypsin-ABSciex) and the reaction was stopped with TFA at a final concentration of 1%. Samples were dried in a Speed Vac, resuspended in 50 µL of TFA (0.1%), and the peptides were concentrated and purified using ZipTip C18 Tips (Merck Millipore, Darmstadt, Germany). Finally, the samples were spiked with a mix of heavy-labeled peptides in 2% ACN and 0.1% FA for injection.

#### SRM-MS

SRM experiments were performed on a 5500 QTRAP hybrid triple quadrupole/linear ion trap mass spectrometer (ABSciex) equipped with an Eksigent 1D + plus nanoLC chromatographic system. Digested samples (1 µg of protein and 25 fmol of each heavy-labeled peptide) were injected onto an Acclaim PepMap100 C18 trap column (100 um × 2 cm, Thermo scientific) and then separated by RP-HPLC on an Acclaim PepMap RSLC C18 column (75 um × 15 cm, Thermo scientific). Chromatography was performed with solvent A (0.1% FA) and solvent B (100% ACN, 0.1% FA) as a mobile phase, using at a 300 nl/min flow rate and a linear gradient: 60 minutes from 2% B to 40% B; 5 minutes to 50% B; and 5 minutes at 90% B before returning to 2% B).

SRM data were acquired in positive mode with: a spray voltage of 2800 V; curtain gas 20 psi; ion source gas 20 psi; interface heater temperature (IHT) 150 °C; DP 80; entrance potential 10; exit potential (EXP) 15; and a pause time of 3 ms. Collision Energy (CE) was estimated using MRM Pilot software (ABsciex) and set according to Table S2. Transitions were monitored using Unit Resolution in both Q1 and Q3 quadrupoles and a 20 ms dwell time for each one. The data was analyzed and the area ratio (light/heavy) for all transitions was calculated using Analyst® 1.5.2 and MultiQuant® 2.0.2 software (ABsciex).

### Statistical analysis

Data are presented as the mean ± standard deviation (SD) or as percentages. For the TMT results, we considered proteins differentially expressed with log2-ratios expressed in the form of the standardized variables (Zq) ±1.5 (p-values ≤ 0.05). The changes in peptide and protein abundance were assessed with a 1% FDR. Comparisons between groups were performed with a Student’s *t*-test (Table 2) or by one-way ANOVA (Table 1). A Pearson’s correlation coefficient was calculated to analyze the association between two variables and a receiver operating characteristic (ROC) analysis was performed using SPSS 15.0.Statistical significance was accepted at *p < 0.05, **p < 0.01, ***p < 0.001.

## Electronic supplementary material

Supplementary Information
