# Supplementary material for: Potential role of new molecular plasma signatures on cardiovascular risk stratification in asymptomatic individuals
Source: Sci Rep. 2018 Mar 19;8:4802. doi: 10.1038/s41598-018-23037-7 (PMC5859270; doi:10.1038/s41598-018-23037-7)
Supplement: Supplementary file 1 — Supplementary Information [file 41598_2018_23037_MOESM1_ESM.doc]

**SUPPLEMENTARY MATERIAL**

**Potential role of new molecular plasma signatures on cardiovascular risk stratification in asymptomatic individuals**

Montserrat Baldan-Martin^1*^, Juan A. Lopez^2*^, Nerea Corbacho^1*^, Paula J. Martinez^3^, Elena Rodriguez-Sanchez^4^, Laura Mourino-Alvarez^1^, Tamara Sastre-Oliva^1^, Tatiana Martin-Rojas^1^, Raul Rincón^1^, Eva Calvo^5^, Jesus Vazquez^2^, Fernando Vivanco^3,6^, Luis R. Padial^7^, Gloria Alvarez-Llamas^3^, Gema Ruiz-Hurtado^4^, Luis M. Ruilope^4,8,9^, Maria G. Barderas^1^.

^1^ Department of Vascular Physiopathology, Hospital Nacional de Paraplejicos (HNP), SESCAM, Toledo, Spain.

^2^ Cardiovascular Proteomics Laboratory and CIBER-CV, CNIC, Madrid, Spain.

^3^ Departament of Immunology, IIS-Fundacion Jimenez Diaz, Madrid, Spain.

^4^ Laboratory of Hypertension and Cardiovascular Risk, Instituto de Investigación i+12, Hospital Universitario 12 de Octubre, Madrid, Spain.

^5^ Ibermutuamur, Madrid, Spain.

^6^ Departamento de Bioquimica y Biologia Molecular I, Universidad Complutense, Madrid, Spain.

^7^ Departamento de Cardiologia, Complejo Hospitalario de Toledo, SESCAM, Toledo, Spain.

^8^Department of Preventive Medicine and Public Health, School of Medicine, Universidad Autónoma de Madrid/IdiPAZ and CIBER in Epidemiology and Public Health (CIBERESP), Madrid, Spain.

^9^School of Doctoral Studies and Research, Universidad Europea de Madrid, Spain.

**^*^**These authors contributed equally to this work.

|  |  |  | **Healthy controls** | **CV risk factor** | **CV event** | **Student´s t-test (p-value)** | | | |
| --- | --- | --- | --- | --- | --- | --- | --- | --- | --- |
| **FASTA** | **Protein** | **Description** | **Mean Zq** | **Mean Zq** | **Mean Zq** | | **CV risk factor/Controls** | **CV event/CV risk factor** | **CV event /Controls** |
| P00739 | HPTR | Haptoglobin-related protein | -6.5 | -4.1 | 3.7 | | 0.3890 | 0.0382 | 0.0022 |
| P02763 | A1AG1 | Alpha-1-acid glycoprotein 1 | -2.0 | 0.5 | 3.3 | | 0.1337 | 0.2839 | 0.0272 |
| P00738 | HPT | Haptoglobin | -4.4 | -4.6 | 3.2 | | 0.9428 | 0.0151 | 0.0005 |
| P62328 | TYB4 | Thymosin beta-4 | -6.7 | -6.4 | 2.9 | | 0.7334 | 0.0013 | 0.0002 |
| P04004 | VTNC | Vitronectin | -1.6 | -3.3 | 2.0 | | 0.0283 | 0.0020 | 0.0037 |
| P02765 | FETUA | Alpha-2-HS-glycoprotein | -1.6 | -3.6 | 2.0 | | 0.0060 | 0.0003 | 0.0035 |
| P04217 | A1BG | Alpha-1B-glycoprotein | -0.9 | -2.0 | 1.8 | | 0.0318 | 0.0002 | 0.0018 |
| P00751 | CFAB | Complement factor B | -1.3 | -1.6 | 1.6 | | 0.7260 | 0.0182 | 0.0056 |
| P01042 | KNG1 | Kininogen-1 | -1.0 | -3.9 | 1.4 | | 0.0114 | 0.0017 | 0.0040 |
| Q9H299 | SH3L3 | SH3 domain-binding glutamic acid-rich-like protein 3 | -1.0 | -2.8 | 1.4 | | 0.0095 | 0.0002 | 0.0047 |
| P06753-6 | TPM3 | Isoform 6 of Tropomyosin alpha-3 chain | -1.5 | -1.7 | 1.2 | | 0.7483 | 0.0034 | 0.0006 |
| P02749 | APOH | Beta-2-glycoprotein 1 | -1.2 | -4.2 | 1.1 | | 0.0267 | 0.0149 | 0.0648 |
| P02753 | RET4 | Retinol-binding protein 4 | -0.4 | -4.2 | 1.0 | | 0.0051 | 0.0033 | 0.0223 |
| P00747 | PLMN | Plasminogen | -0.3 | -4.0 | 0.5 | | 0.0074 | 0.0074 | 0.2514 |
| P00740 | FA9 | Coagulation factor IX | -0.2 | -3.2 | 0.4 | | 0.0031 | 0.0024 | 0.2795 |
| P31944 | CASPE | Caspase-14 | 0.3 | 3.3 | -0.2 | | 0.0115 | 0.0021 | 0.5409 |
| P15169 | CBPN | Carboxypeptidase N catalytic chain | 1.8 | 1.8 | -0.2 | | 0.9955 | 0.0139 | 0.0359 |
| P05089 | ARGI1 | Arginase-1 | 0.8 | 3.5 | -0.5 | | 0.0253 | 0.0102 | 0.2196 |
| P20930 | FILA | Filaggrin | -0.2 | 5.6 | -0.5 | | 0.0422 | 0.0611 | 0.7858 |
| Q02413 | DSG1 | Desmoglein-1 O | 0.5 | 4.7 | -0.7 | | 0.0094 | 0.0058 | 0.2886 |
| P15924 | DESP | Desmoplakin | 1.2 | 9.0 | -0.8 | | 0.0046 | 0.0027 | 0.1702 |
| P02790 | HEMO | Hemopexin | 0.9 | -3.2 | -0.9 | | 0.0065 | 0.0219 | 0.1729 |
| P14923 | PLAK | Junction plakoglobin | 1.3 | 6.2 | -1.1 | | 0.0405 | 0.0150 | 0.1489 |
| P81605 | DCD | Dermcidin | -0.6 | 2.9 | -1.1 | | 0.0078 | 0.0151 | 0.6804 |
| P01011 | AACT | Alpha-1-antichymotrypsin | 1.5 | 1.3 | -1.5 | | 0.7982 | 0.0812 | 0.0206 |
| P35858 | ALS | Insulin-like growth factor-binding protein complex acid labile subunit | 2.3 | 1.7 | -1.6 | | 0.4363 | 0.0119 | 0.0017 |
| Q15582 | BGH3 | Transforming growth factor-beta-induced protein ig-h3 | 2.7 | 1.5 | -1.7 | | 0.0163 | 0.0137 | 0.0011 |
| Q86UX7 | URP2 | Fermitin family homolog 3 | 0.5 | 0.0 | -1.7 | | 0.4583 | 0.0273 | 0.0122 |
| P23284 | GSTO1 | Glutathione S-transferase omega-1 | 1.5 | 1.0 | -1.8 | | 0.2108 | 0.0012 | 0.0005 |
| P23284 | PPIB | Peptidyl-prolylcis-trans isomerase B | 1.4 | 0.7 | -1.8 | | 0.4000 | 0.0224 | 0.0078 |
| Q86YZ3 | HORN | Hornerin | -0.9 | 11.2 | -2.0 | | 0.0112 | 0.0147 | 0.5822 |
| Q9HBI1 | PARVB | Beta-parvin | 1.6 | 2.8 | -2.1 | | 0.1572 | 0.0001 | 0.0006 |
| P06276 | CHLE | Cholinesterase | 2.1 | 2.0 | -2.1 | | 0.9795 | 0.0048 | 0.0029 |
| P01033 | TIMP1 | Metalloproteinase inhibitor 1 | 1.6 | 0.9 | -2.2 | | 0.2403 | 0.0003 | 0.0003 |
| P84077 | ARF1 | ADP-ribosylation factor 1 | 1.5 | 1.7 | -2.2 | | 0.7333 | 0.0009 | 0.0014 |
| P68104 | EF1A1 | Elongation factor 1-alpha 1 | 1.3 | 2.5 | -2.4 | | 0.1863 | 0.0001 | 0.0024 |
| Q96KN2 | CNDP1 | Beta-Ala-His dipeptidase | 1.9 | 0.9 | -2.4 | | 0.2107 | 0.0566 | 0.0090 |
| Q9H4M9 | EHD1 | EH domain-containing protein 1 | 1.4 | 1.7 | -2.5 | | 0.6113 | 0.0004 | 0.0002 |
| P20851 | C4BPB | C4b-binding protein beta chain | 2.6 | 2.2 | -2.6 | | 0.7586 | 0.0037 | 0.0018 |
| P05155 | IC1 | Plasma protease C1 inhibitor | 2.7 | 2.9 | -2.7 | | 0.7853 | 0.0137 | 0.0077 |
| P02679 | FIBG | Fibrinogen gamma chain | 2.0 | 1.8 | -2.8 | | 0.8277 | 0.0029 | 0.0002 |
| P04114 | APOB | Apolipoprotein B-100 | 2.1 | 1.2 | -2.9 | | 0.3553 | 0.0620 | 0.0080 |
| Q5D862 | FILA2 | Filaggrin-2 | 2.0 | 5.0 | -3.0 | | 0.0804 | 0.0004 | 0.0042 |
| P02655 | APOC2 | Apolipoprotein C-II | 0.0 | 5.6 | -3.3 | | 0.0025 | 0.0003 | 0.0246 |
| P02649 | APOE | Apolipoprotein E | 2.4 | 5.9 | -3.9 | | 0.1160 | 0.0018 | 0.0012 |
| P06727 | APOA4 | Apolipoprotein A-IV | 4.4 | 3.1 | -6.0 | | 0.1025 | 0.0006 | 0.0001 |
| P02647 | APOA1 | Apolipoprotein A-I | 5.5 | 4.8 | -6.8 | | 0.8315 | 0.0148 | 0.0009 |

**Table S1: List of 47 proteins differentially expressed between different groups of studies by TMT 10-plex analysis**. Using student´s t test, p-value <0.05 was considered statistically significant.

| **PROTEIN ID** | **Q1 (m/z)** | **Q3 (m/z)** | **CE** |
| --- | --- | --- | --- |
| C4BPB-P20851.ALLAFQESK.2/y6.light | 503.78 | 709.35 | 27.2 |
| C4BPB-P20851.ALLAFQESK.2/y5.light | 503.78 | 638.31 | 27.2 |
| C4BPB-P20851.ALLAFQESK.2/y7.light | 503.78 | 822.44 | 27.2 |
| C4BPB-P20851.ALLAFQESK.2/y5.heavy | 507.79 | 646.33 | 27.3 |
| C4BPB-P20851.ALLAFQESK.2/y6.heavy | 507.79 | 717.37 | 27.3 |
| C4BPB-P20851.ALLAFQESK.2/y7.heavy | 507.79 | 830.45 | 27.3 |
| CFAB-P00751.YGLVTYATYPK.2/y7.light | 638.33 | 843.42 | 33.1 |
| CFAB-P00751.YGLVTYATYPK.2/y8.light | 638.33 | 942.49 | 33.1 |
| CFAB-P00751.YGLVTYATYPK.2/y9.light | 638.33 | 1055.58 | 33.1 |
| CFAB-P00751.YGLVTYATYPK.2/y7.heavy | 642.34 | 851.44 | 33.3 |
| CFAB-P00751.YGLVTYATYPK.2/y8.heavy | 642.34 | 950.51 | 33.3 |
| CFAB-P00751.YGLVTYATYPK.2/y9.heavy | 642.34 | 1063.59 | 33.3 |
| RET4-P02753.YWGVASFLQK.2/y6.light | 599.82 | 693.39 | 31.4 |
| RET4-P02753.YWGVASFLQK.2/y7.light | 599.82 | 792.46 | 31.4 |
| RET4-P02753.YWGVASFLQK.2/y8.light | 599.82 | 849.48 | 31.4 |
| RET4-P02753.YWGVASFLQK.2/y9.light | 599.82 | 1035.56 | 31.4 |
| RET4-P02753.YWGVASFLQK.2/y6.heavy | 603.82 | 701.41 | 31.6 |
| RET4-P02753.YWGVASFLQK.2/y7.heavy | 603.82 | 800.48 | 31.6 |
| RET4-P02753.YWGVASFLQK.2/y8.heavy | 603.82 | 857.5 | 31.6 |
| RET4-P02753.YWGVASFLQK.2/y9.heavy | 603.82 | 1043.58 | 31.6 |
| VTNC-P04004.GQYC[CAM]YELDEK.2/y6.light | 652.78 | 796.37 | 33.7 |
| VTNC-P04004.GQYC[CAM]YELDEK.2/y5.light | 652.78 | 633.31 | 33.7 |
| VTNC-P04004.GQYC[CAM]YELDEK.2/y7.light | 652.78 | 956.4 | 33.7 |
| VTNC-P04004.GQYC[CAM]YELDEK.2/y8.light | 652.78 | 1119.47 | 33.7 |
| VTNC-P04004.GQYC[CAM]YELDEK.2/y5.heavy | 656.78 | 641.32 | 33.9 |
| VTNC-P04004.GQYC[CAM]YELDEK.2/y6.heavy | 656.78 | 804.39 | 33.9 |
| VTNC-P04004.GQYC[CAM]YELDEK.2/y7.heavy | 656.78 | 964.42 | 33.9 |
| VTNC-P04004.GQYC[CAM]YELDEK.2/y8.heavy | 656.78 | 1127.48 | 33.9 |
| CNDP1-Q96KN2.ALEQDLPVNIK.2/y5.light | 620.35 | 570.36 | 32.3 |
| CNDP1-Q96KN2.ALEQDLPVNIK.2/y6.light | 620.35 | 683.45 | 32.3 |
| CNDP1-Q96KN2.ALEQDLPVNIK.2/y7.light | 620.35 | 798.47 | 32.3 |
| CNDP1-Q96KN2.ALEQDLPVNIK.2/y9.light | 620.35 | 1055.57 | 32.3 |
| CNDP1-Q96KN2.ALEQDLPVNIK.2/y5.heavy | 624.36 | 578.38 | 32.5 |
| CNDP1-Q96KN2.ALEQDLPVNIK.2/y6.heavy | 624.36 | 691.46 | 32.5 |
| CNDP1-Q96KN2.ALEQDLPVNIK.2/y7.heavy | 624.36 | 806.49 | 32.5 |
| CNDP1-Q96KN2.ALEQDLPVNIK.2/y9.heavy | 624.36 | 1063.59 | 32.5 |
| CBPN-P15169.IVQLIQDTR.2/y5.light | 543.32 | 632.34 | 28.9 |
| CBPN-P15169.IVQLIQDTR.2/y6.light | 543.32 | 745.42 | 28.9 |
| CBPN-P15169.IVQLIQDTR.2/y7.light | 543.32 | 873.48 | 28.9 |
| CBPN-P15169.IVQLIQDTR.2/y5.heavy | 548.32 | 642.34 | 29.1 |
| CBPN-P15169.IVQLIQDTR.2/y6.heavy | 548.32 | 755.43 | 29.1 |
| CBPN-P15169.IVQLIQDTR.2/y7.heavy | 548.32 | 883.49 | 29.1 |
| TPM3-P06753.IQLVEEELDR.2/y5.light | 622.33 | 661.32 | 32.4 |
| TPM3-P06753.IQLVEEELDR.2/y6.light | 622.33 | 790.36 | 32.4 |
| TPM3-P06753.IQLVEEELDR.2/y7.light | 622.33 | 889.43 | 32.4 |
| TPM3-P06753.IQLVEEELDR.2/y5.heavy | 627.33 | 671.32 | 32.6 |
| TPM3-P06753.IQLVEEELDR.2/y6.heavy | 627.33 | 800.37 | 32.6 |
| TPM3-P06753.IQLVEEELDR.2/y7.heavy | 627.33 | 899.43 | 32.6 |
| ApoA-IV-P06727.LGEVNTYAGDLQK.2/y8.light | 704.36 | 895.45 | 36.0 |
| ApoA-IV-P06727.LGEVNTYAGDLQK.2/y9.light | 704.36 | 1009.49 | 36.0 |
| ApoA-IV-P06727.LGEVNTYAGDLQK.2/y10.light | 704.36 | 1108.56 | 36.0 |
| ApoA-IV-P06727.LGEVNTYAGDLQK.2/y8.heavy | 708.37 | 903.47 | 36.2 |
| ApoA-IV-P06727.LGEVNTYAGDLQK.2/y9.heavy | 708.37 | 1017.51 | 36.2 |
| ApoA-IV-P06727.LGEVNTYAGDLQK.2/y10.heavy | 708.37 | 1116.58 | 36.2 |

**Table S2. SRM assay developed for measuring the expression of proteins differentially expressed in discovery phase.** Q1: m/z selected in the first quadrupole (precursor); Q3: m/z selected in the third quadrupole (product) and the corresponding collision energy (CE).
